# Pharmacological inhibitors of TRPV4 channels reduce cytokine production, restore endothelial function and increase survival in septic mice

**DOI:** 10.1038/srep33841

**Published:** 2016-09-22

**Authors:** Thomas Dalsgaard, Swapnil K. Sonkusare, Cory Teuscher, Matthew E. Poynter, Mark T. Nelson

**Affiliations:** 1 Department of Pharmacology, College of Medicine, University of Vermont, Burlington, VT 05405, USA; 2 Department of Medicine, Immunobiology Program, University of Vermont, Burlington, VT 05405, USA; 3 Department of Medicine, Division of Pulmonary Disease and Critical Care, University of Vermont, Burlington, VT 05405, USA; 4 Institute of Cardiovascular Sciences, University of Manchester, Manchester M13 9NT, UK

## Abstract

Sepsis is characterized by systemic inflammation, edema formation and hypo-perfusion leading to organ dysfunction and ultimately death. Activation of the transient receptor potential vanilloid type 4 (TRPV4) channel is associated with edema formation and circulatory collapse. Here, we show that TRPV4 channels are involved in the hyper-inflammatory response and mortality associated with sepsis. Pharmacological inhibition of TRPV4 channels in mice reduced mortality in lipopolysaccharide and cecal-ligation-and-puncture models of sepsis, but not in a tumor necrosis factor-α (TNFα)-induced sepsis model. These protective effects of TRPV4 channel inhibition were attributable to prevention of the sepsis-induced surge of a broad spectrum of pro-inflammatory cytokines, including TNFα, interleukin (IL)-1 and IL-6, and subsequent preservation of endothelial cell function, including Ca^2+^ signaling, integrity and endothelium-dependent vasodilation. These results suggest that TRPV4 antagonists may be of therapeutic utility in the management of sepsis.

Severe sepsis and septic shock are significant and growing causes of morbidity and mortality. There are approximately 850,000 cases of sepsis annually in the United States, and the associated health care costs are high (~$20 billion/year). Despite treatment, the septic shock–associated mortality is 47%^1^,^2^,^3^.

Sepsis is a multifactorial process characterized by a systemic inflammatory response to infection. Considerable research effort has been devoted to understanding the complex and dynamic pathophysiological mechanisms that underlie the heterogeneous sepsis syndrome, as reflected in the substantial body of literature on the subject. Sepsis develops when the initial appropriate host response to an infection becomes amplified and subsequently dysregulated^4^. Hyper-activation of this cellular defense results in excessive release of cytokines, chemokines, and other inflammatory regulators. Immune and inflammatory responses are tightly integrated with other systemic physiological processes, including coagulation^5^, metabolism^6^, and neuroendocrine activation^7^,^8^. For instance, inflammation-induced dysregulation of the coagulation system significantly aggravates the deleterious effects of sepsis and can result in lethal disseminated intravascular coagulation (DIC)^9^. The hyper-inflammatory phase, which is initiated by tumor necrosis factor-α (TNFα) and subsequently amplified by interleukin-1 (IL-1) and IL-6^4^,^10^, may, together with downstream mediators, lead to endothelial dysfunction, characterized by vasodilation and increased permeability. The resulting vascular leakage syndrome is clinically associated with hypotension and edema^11^, and ultimately causes remote organ dysfunction and death^12^. Many potential therapeutic options for sepsis have failed in the clinic. This failure has been attributed to the complexity of the inflammatory cytokine and coagulation cascades, which consist of overlapping mechanisms of action that cannot be disabled by targeting one single pathway. For example, neither targeting the pro-inflammatory response with anti-TNFα antibody therapy nor DIC formation with activated protein C has been successful in human sepsis^13^,^14^,^15^,^16^. Thus, there is a need for therapeutic agents capable of targeting multiple processes to provide an effective therapeutic intervention in sepsis.

TRPV4 channels, members of the vanilloid family of the transient receptor potential (TRP) cation channel superfamily, are activated by mechanical stimuli, such as pressure^17^,^18^ and heat^19^,^20^, as well as pharmacological agonists^20^,^21^,^22^,^23^, and may also have a role in osmoregulation^24^,^25^,^26^ and sodium regulation^27^. TRPV4 channels are prominently expressed in vascular endothelial cells, and activation of TRPV4 channels as well as IP_3_ receptors are the major mechanisms that elevate endothelial cell intracellular Ca^2+^. The elevation of intracellular Ca^2+^ in turn causes endothelial-dependent vasodilation through activation of Ca^2+^-sensitive potassium channels and nitric oxide production by nitric oxide synthase^28^,^29^,^30^. Local Ca^2+^ signals are central to the elaboration of these dilatory pathways. Ca^2+^ influx through single TRPV4 channels (Ca^2+^ “sparklet”) has been measured in native endothelial cells and contributes to endothelial-dependent dilation and dysfunction^28^,^29^. Elementary and stationary IP_3_-mediated Ca^2+^ signals (Ca^2+^ “pulsars”) occur primarily at the endothelial projections to smooth muscle, and are also involved in vasoregulation^31^. TRPV4 channels can also regulate vascular permeability *in vivo*, as observed in the lung^22^,^32^,^33^. *In vivo*, excessive activation of TRPV4 channels causes endothelial detachment from the basement membrane, which leads to disruption of the pulmonary endothelial barrier, pulmonary edema, and alveolar flooding^34^,^35^. This TRPV4-induced disruption of the endothelial barrier has been recapitulated in cultured^22^, and more recently in native^28^, endothelial cells. Taken together, these observations indicate that TRPV4 channels can regulate blood pressure and fluid and electrolyte balance—the processes controlling edema formation. In addition to being expressed in vascular cells, TRPV4 channels have also been found at high levels in macrophages^36^,^37^,^38^, where they may be involved in Ca^2+^ regulation and the production of superoxide, nitric oxide^36^, and cytokines^32^ in ventilator- and chemical-induced lung injury.

Despite the provocative association of TRPV4 function with certain key features of sepsis, including systemic inflammatory responses, endothelial dysfunction and subsequent hypotension and edema formation, whether activation of TRPV4 channels is involved in the development of sepsis is unknown. Here, using three different mouse models, we investigated the contribution of TRPV4 channels to the development of sepsis, and sought to determine whether pharmacological blockade of these channels with potent, selective TRPV4 antagonists exerts a protective effect. In this study, we provide the first evidence that inhibition of TRPV4 channels reduces the hyper-inflammatory response in sepsis and increases survival. Specifically, we found that treatment with TRPV4 channel inhibitors reduced mortality in lipopolysaccharide (LPS) and cecal-ligation-and-puncture (CLP), but not in TNFα, models of sepsis in mice. Furthermore, inhibition of TRPV4 channels reduced the *in vivo* levels of pro-inflammatory cytokines and maintained endothelial function, measured as changes in vasodilation and barrier function. We conclude that the TRPV4 channel is a potential new therapeutic target in the critical, early hyper-inflammatory phase of sepsis that precedes pro-inflammatory cytokine production.

## Results

### TRPV4 channel inhibition increases survival in sepsis induced by CLP and LPS, but not TNFα

**Figure 1:**
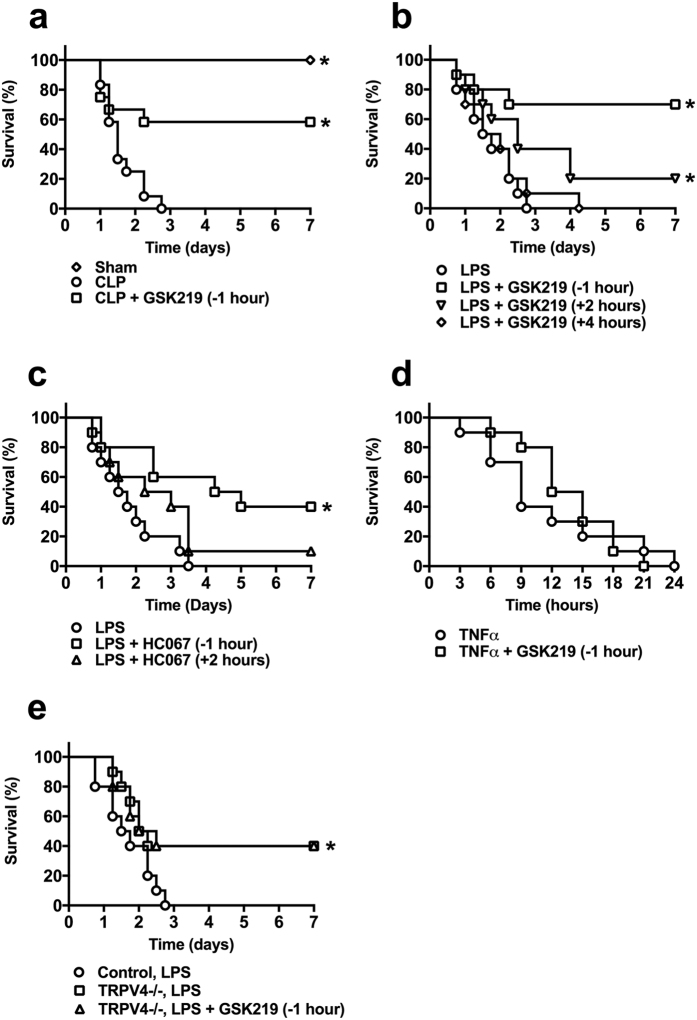
TRPV4 channel blockade protects mice from the lethality of LPS and CLP, but not TNFα, *in vivo*. (**a**) Seven-day survival of CLP model mice, without (CLP) or with (CLP + GSK219) GSK219; GSK219 (1 mg/kg, i.p.) was administered 1 hour prior to CLP (Sham, n = 5; CLP and CLP + GSK219, n = 12; *p < 0.05 vs. CLP alone, Mantel-Cox test). (**b**) Seven-day survival of mice injected with LPS only (50 mg/kg, i.p.) or LPS + GSK219; GSK219 (1 mg/kg, i.p.) was administered 1 hour prior to or 2 or 4 hours after LPS injection (n = 10 mice/group; *p < 0.05 vs. LPS treatment, Mantel-Cox test). (**c**) Seven-day survival of mice injected with LPS only (50 mg/kg, i.p.) or LPS + HC067; HC067 (10 mg/kg, i.p.) was administered 1 hour prior to or 2 hours after LPS injection (n = 10 mice/group; *p < 0.05 vs. LPS treatment, Mantel-Cox test). (**d**) Twenty-four-hour survival of mice injected with TNFα only (1 mg/kg, i.p.) or with TNFα + GSK219; GSK219 (1 mg/kg, i.p.) was administered 1 hour prior to TNFα injection (n = 10 mice/group; *p < 0.05 vs. TNFα treatment, Mantel-Cox test). (**e**) Seven-day survival of normal or TRPV4−/− mice injected with LPS only (50 mg/kg, i.p.) or TRPV4−/− mice injected with LPS + GSK219. GSK219 (1 mg/kg, i.p.) was administered 1 hour prior to LPS injection (n = 10 mice/group; *p < 0.05 vs. LPS treatment, Mantel-Cox test).

To evaluate the role of TRPV4 channels in sepsis, we used the highly selective inhibitors, GSK2193874 (hereafter GSK219)^33^ and HC067047 (hereafter HC067)^28^,^39^, which have been shown to be well tolerated *in vivo*, with minimal side effects^33^,^39^. As a first approach for inducing sepsis, we employed the widely used polymicrobial sepsis model, cecal ligation and puncture (CLP)^40^. Mice subjected to CLP and administered vehicle (DMSO) died within 3 days, with a median survival of 1.5 days. In contrast, 60% of mice that received the TRPV4 antagonist, GSK219, 1 hour prior to the CLP surgery survived the entire 7-day observation period and appeared normal after day 4 (Fig. 1a).

Despite the widespread use of CLP as an experimental model of sepsis, the surgical technique involved creates challenges for consistently obtaining reliable and reproducible results^40^. We therefore extended these studies using the experimentally simpler, and thus more reproducible, LPS and TNFα models, created by injecting mice with LPS (50 mg/kg, i.p.) or TNFα (1 mg/kg, i.v.). Although these sepsis models do not include the polymicrobial aspect of sepsis, they have very similar pathophysiology in terms of inflammatory response, vascular dysfunction, septic shock, and multi-organ dysfunction^41^,^42^,^43^. The effects of TRPV4 inhibition were tested by treating mice with GSK219 (1 mg/kg, i.p.) or HC067 (10 mg/kg, i.p.) 1 hour before or at different times after LPS or TNFα injection. In the LPS-induced sepsis model, the median survival of mice that were not treated with a TRPV4 antagonist was 1.6 days, and no mice survived more than 3 days after LPS injection (Fig. 1b,c). In contrast, the survival rate was dramatically increased by pretreating mice with GSK219 1 hour prior to LPS injection; under these conditions, 70% of mice survived the entire 7-day observation period (Fig. 1b). GSK219 pretreatment also attenuated the LPS-induced increase in the levels of blood urea nitrogen (BUN), a kidney injury marker (Suppl. Fig. 1e), but did not affect LPS-induced increases in creatinine (Suppl. Fig. 1f) or the liver injury markers aspartate aminotransferase (AST) (Suppl. Fig. 1c) and alanine aminotransferase (ALT) (Suppl. Fig. 1a). Pretreatment with the antagonist HC067 similarly protected against mortality, producing a median survival of 4.6 days and a survival rate of 40% at 7 days (Fig. 1c).

To determine if post-LPS inhibition of TRPV4 channels afforded protection against sepsis, we examined survival rates in the LPS model in mice administered a TRPV4 antagonist 2 or 4 hours after LPS. GSK219 administered 2 hours after LPS injection improved survival (Fig. 1b), but had no protective effect if given after 4 hours (Fig. 1b). Treatment with the less potent TRPV4 antagonist HC067 2 hours after LPS was not protective (median survival = 2.6 days; 7-day survival = 10%) (Fig. 1c). In contrast to the protective effects observed in the CLP and LPS models, pretreatment with GSK219 had no effect in the TNFα sepsis model; mice in both GSK219-treated and -untreated groups survived 24 hours or less (Fig. 1d). Supporting an essential role for TRPV4 channels in the development of sepsis, TRPV4−/− mice treated with LPS had higher survival rates (median survival = 2.2 days; 7-day survival = 40%) compared to normal mice (median survival = 1.6 days; 7-day survival = 0%). Furthermore, administering GSK219 to TRPV4−/− mice prior to LPS treatment resulted in no difference in survival from treatment with LPS alone. Taken together, these results indicate that the TRPV4 channels participate in the early events of sepsis development and that TRPV4 channel antagonists confer protection against sepsis-induced mortality when administered early in disease progression. Moreover, the absence of an effect of TRPV4 channel antagonists in the TNFα model of sepsis suggests that blocking TRPV4 does not protect tissues from the actions of TNFα and possibly other pro-inflammatory cytokines that are produced in high concentrations in sepsis, but instead likely modulates critical events preceding cytokine production during the initial pro-inflammatory phase of sepsis.

### TRPV4 channel inhibition reduces cytokine levels in sepsis

**Figure 2:**
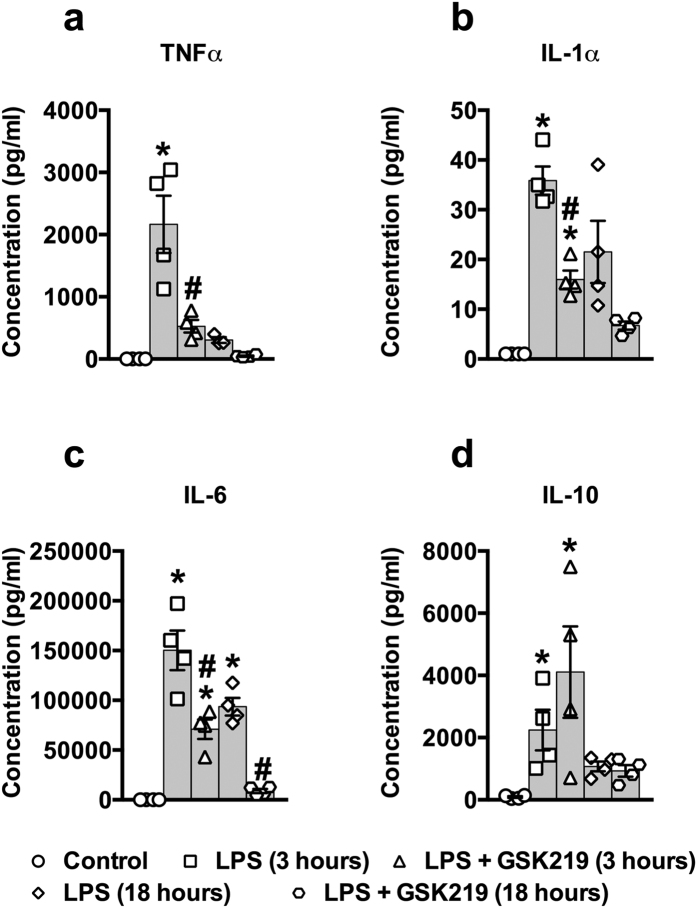
TRPV4 channel blockade reduces the concentration of the pro-inflammatory cytokines, TNFα, IL-1α, and IL-6, but not the anti-inflammatory cytokine, IL-10, in LPS-induced sepsis *in vivo*. Blood concentrations of (**a**) TNFα, (**b**) IL-1α, (**c**) IL-6, and (**d**) IL-10, 3 and 18 hours after injection of LPS only (50 mg/kg, i.p.) or LPS + GSK219 (1 mg/kg, i.p., injected 1 hour prior to LPS). Data are expressed as means ± SEM (n = 4 mice/group; *p < 0.05 vs. control, ^#^p < 0.05 vs. LPS treatment at the same time point, Kruskal-Wallis test with Dunn’s multiple comparisons test). IL, interleukin; TNFα, tumor necrosis factor alpha.

Sepsis induces a massive release of pro-inflammatory cytokines, which impact key organs in disease progression. We therefore tested the effects of the TRPV4 antagonist GSK219 on the levels of sepsis-induced circulating cytokines, measuring serum concentrations 3 and 18 hours after LPS injection in control (LPS alone) and GSK219-pretreated (LPS + GSK219) mice. Administration of GSK219 blunted the LPS-induced increase in a large number of pro-inflammatory cytokines, including TNFα, IL-1α, and IL-6, cytokines suggested to be key mediators of the hyper-inflammatory response in sepsis (Fig. 2a–c), as well as eotaxin, GM-CSF (granulocyte/monocyte colony-stimulating factor), interferon (IFN)-γ, IL-1β, IL-3, IL-5, IL-12p40, IL-12p70, IL-13, IL-17α, MCP-1, MIP-1α, MIP-1β, RANTES (Suppl. Fig. 2), at one or both time points. Furthermore, TNFα and IL-6 production by peritoneal leukocytes isolated from GSK219 pretreated mice 1 hour after LPS-injection and cultured in normal medium were also reduced compared to that by cells from mice exposed *in vivo* to LPS alone (Suppl. Fig. 3), indicating that the effects of TRPV4 channel blockade persist *ex vivo*. Interestingly, the anti-inflammatory cytokine IL-10 was unaffected by GSK210 treatment (Fig. 2d). These results clearly show that TRPV4 channel activity is an essential part of the hyper-inflammatory response observed in sepsis and further indicate that blocking TRPV4 channels substantially reduces the production of cytokines, not only at the beginning of the development of sepsis, but also at a later time point.

### TRPV4 channel inhibition protects against endothelial dysfunction in sepsis

To determine if inhibition of TRPV4 channels protects against cytokine-induced endothelial dysfunction, we measured the abundance of circulating factors indicative of a general level of endothelial activation—a pro-inflammatory and pro-coagulant endothelial cell state that promotes endothelial dysfunction—in mice treated with LPS or LPS+GSK219.

**Figure 3:**
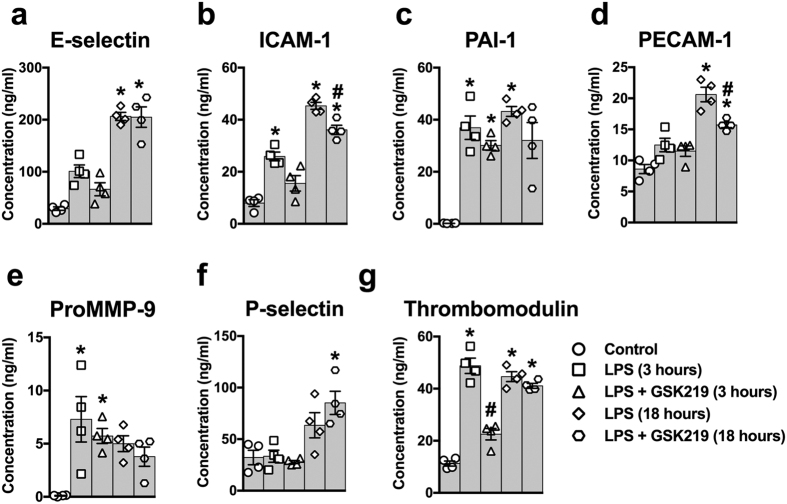
TRPV4 channel blockade reduces the concentration of blood markers of activated vascular endothelium in LPS-induced sepsis *in vivo*. Blood concentrations of soluble (**a**) E-selectin, (**b**) ICAM-1, (**c**) PAI-1, (**d**) PECAM-1, (**e**) ProMMP-9, (**f**) P-selectin and (**g**) thrombomodulin, 3 and 18 hours after injection of LPS only (50 mg/kg, i.p.) or LPS + GSK219 (1 mg/kg, i.p., injected 1 hour prior to LPS). Data are expressed as means ± SEM (n = 4 mice/group; *p < 0.05 vs. control, ^#^p < 0.05 vs. LPS treatment at the same time point, Kruskal-Wallis test with Dunn’s multiple comparisons test). ICAM, intercellular adhesion molecule; PAI, plasminogen activator inhibitor; PECAM, platelet endothelial cell adhesion molecule; MMP, matrix metallopeptidase.

Of the seven endothelial cell activation markers investigated (E-selectin, intercellular adhesion molecule-1 (ICAM-1), plasminogen activator inhibitor-1 (PAI-1), platelet endothelial cell adhesion molecule-1 (PECAM-1), pro-matrix metallopeptidase 9 (ProMMP9), P-selectin, and thrombomodulin) (Fig. 3), we found that administration of the TRPV4 channel antagonist GSK219 blunted the LPS-induced increases in ICAM-1 (Fig. 3b) and PECAM-1 (Fig. 3d), which primarily facilitate migration of leukocytes across the endothelium, as well as the anticoagulant thrombomodulin (Fig. 3g).

**Figure 4:**
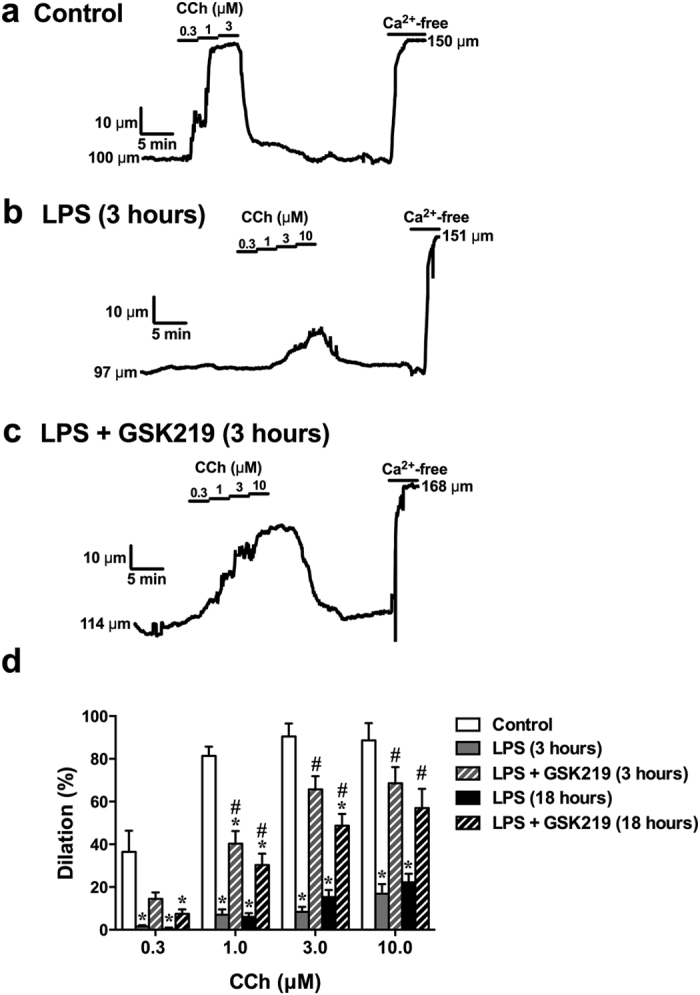
TRPV4 channel blockade prevents LPS-induced endothelial dysfunction in isolated mesenteric arteries. Vasodilation responses in isolated, pressurized (80 mmHg) mesenteric arteries from mice treated with LPS only or LPS + GSK219 measured 3 and 18 hours after LPS. In all cases, LPS and GSK219 were injected (i.p.) at doses of 50 mg/kg and 1 mg/kg, respectively; where used, GSK219 was administered 1 hour prior to LPS injection. (**a–c**) CCh-induced dilation in a control mouse (**a**), a mouse treated with LPS only, measured after 3 hours (**b**); a mouse treated with LPS + GSK219, measured after 3 hours (**c**). (**d**) Summary figure showing quantitative analyses of dilations to CCh (0.3–10 μM) 3 and 18 hours after treatment with LPS only or LPS + GSK219. Arteries were treated with Ca^2+^-free PSS at the conclusion of each experiment to obtain maximal dilation. Data are expressed as mean ± SEM (n = 5 mice/group; *p < 0.05 vs. control, ^#^p < 0.05 vs. LPS treatment at the same time point, Kruskal-Wallis test with Dunn’s multiple comparisons test. CCh, carbachol; PSS, physiological saline solution.

Another characteristic of endothelial dysfunction in sepsis is blunted endothelial-dependent vasodilation and increased vascular permeability—primary causes of hypo-perfusion and edema formation. Figure 4(a–c) shows representative traces of changes in vascular diameter in response to the endothelium-dependent vasodilator carbachol (CCh) in mesenteric arteries isolated from control mice and mice treated with LPS only or LPS + GSK219. In mesenteric arteries from control mice (Fig. 4a), CCh induced maximal dilation at a concentration of 1 μM (81% ± 4%) (Fig. 4a,d). In contrast, in mesenteric arteries from LPS-treated mice, endothelial-dependent vasodilation was dramatically diminished after 3 hours, even at 10 μM CCh (17% ± 5%) (Fig. 4b,d), an effect that persisted at 18 hours after injection (22% ± 4% at 10 μM CCh) (Fig. 4d). In mesenteric arteries from LPS+GSK219–treated mice, CCh-induced dilations to 10 μM CCh were largely restored after 3 (69% ± 8%) and 18 (57% ± 9%) hours (Fig. 4c,d).

**Figure 5:**
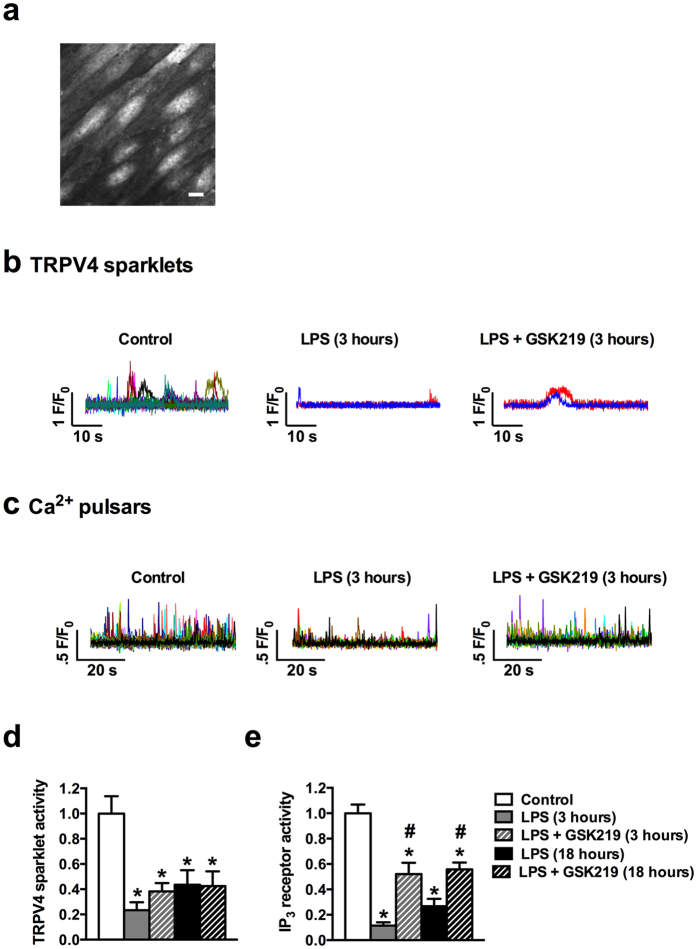
TRPV4 channel blockade attenuates LPS-induced decreases in vascular endothelial TRPV4 sparklets and IP_3_-mediated Ca^2+^ pulsars. Recordings of TRPV4 sparklets and IP_3_-mediated Ca^2+^ pulsars (measures of Ca^2+^-dependent endothelial cell vasoregulatory activity) in Fluo-4–loaded *en face* mesenteric arteries over a 60-second time period. (**a**) Grayscale image showing the endothelial cells in a typical field of view. Scale bar represents 10 μm. (**b,c**) Representative traces showing changes in Fluo-4 fluorescence caused by TRPV4 sparklets and IP_3_-mediated pulsars. Each colored trace represents a different region of interest from an *en face* preparation showing TRPV4 (**b**) and IP_3_ (**c**) Ca^2+^ events in mesenteric arteries from a control mouse; a mouse treated with LPS only, measured after 3 hours; and a mouse treated with LPS + GSK219, measured after 3 hours. In all cases, LPS and GSK219 were injected (i.p.) at doses of 50 mg/kg and 1 mg/kg, respectively; where used, GSK219 was administered 1 hour prior to LPS injection. (**d,e**) Summary figure showing relative TRPV4 (**d**) and IP_3_ (**e**) Ca^2+^ activity 3 and 18 hours after treatment with LPS only and LPS + GSK219. Data are expressed as mean ± SEM (n = 6 mice/group; *p < 0.05 vs. control, ^#^p < 0.05 vs. LPS treatment at the same time point, Kruskal-Wallis test with Dunn’s multiple comparisons test). IP3, inositol trisphosphate.

**Figure 6:**
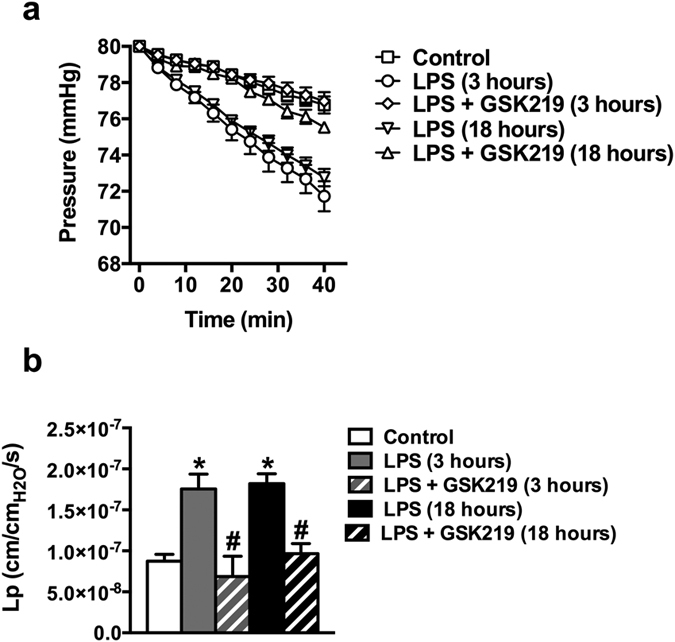
TRPV4 channel blockade protects against LPS-induced increases in mesenteric artery permeability. Pressurized (80 mmHg) mesenteric arteries, isolated from control mice and mice treated with LPS or LPS + GSK219, 3 and 18 hours after treatment, were subjected to a 40-minute pressure-drop permeability protocol, as described in Materials and Methods. In all cases, LPS and GSK219 were injected (i.p.) at doses of 50 mg/kg and 1 mg/kg, respectively; where used, GSK219 was administered 1 hour prior to LPS injection. (**a**) Pressure values measured as a function of time after disconnecting the pressure-servo controller and peristaltic pump. (**b**) Summary data of average hydraulic conductivity (Lp), a measure of endothelial permeability. Data are expressed as means ± SEM (n = 7 mice/group; *p < 0.05 vs. control, ^#^p < 0.05 vs. LPS treatment at the same time point, Kruskal-Wallis test with Dunn’s multiple comparisons test).

Sparklets and pulsars are key measures of Ca^2+^-dependent endothelial cell vasoregulatory activity^28^,^29^,^31^. In support of the development of endothelial dysfunction in sepsis, LPS injection reduced TRPV4 sparklets and IP_3_-mediated Ca^2+^ pulsars in Fluo-4–loaded *en face* mesenteric arteries (Fig. 5). Pretreatment with GSK219 did not recover endothelial TRPV4 sparklets, but blunted the suppression of endothelial cell Ca^2+^ pulsars 3 and 18 hours after LPS injection (Fig. 5c,e). Since endothelial dysfunction in sepsis also confers increased vascular permeability, we tested the role of TRPV4 channels in this process by measuring hydraulic conductivity (L_p_) in mesenteric arteries. LPS treatment doubled hydraulic conductivity from 0.9 ± 0.1 × 10^−7^ to 1.8 ± 0.2 × 10^−7^ and 1.8 ± 0.1 × 10^−7 ^cm/cm_H2O_/s after 3 and 18 hours, respectively. Notably, pretreatment with GSK219 completely prevented the LPS-induced increase in permeability across the vessel wall (Fig. 6).

Taken together, these results indicate that blocking TRPV4 channels protects against the endothelial dysfunction caused by LPS, by reducing the pro-inflammatory and pro-coagulant state of the endothelium and largely preserving endothelial-dependent vasodilation and barrier function.

## Discussion

In the current study, we found that early treatment with TRPV4 channel inhibitors improved survival and decreased hyper-inflammatory cytokine levels in both LPS and CLP sepsis models. Survival was increased dramatically (60–70%) in both models when mice were treated with the TRPV4 antagonist prior to the induction of sepsis. Further supporting the essential role for TRPV4 channels in sepsis, TRPV4−/− mice treated with LPS also had increased survival rates compared to normal mice. Treating LPS-injected TRPV4−/− mice with GSK219 had no effect on survival, indicating that the TRPV4 channel inhibitor acts specifically on TRPV4 channels. Taken together, these results suggest that TRPV4 channel antagonists exert their protective effect in part by reducing the levels of the hyper-inflammatory cytokines involved in the initial phase of sepsis development. Consistent with the interpretation that TRPV4 channel blockade prevents cytokine elaboration instead of protecting target tissues from the deleterious effects of cytokines, we found that TRPV4 antagonism did not increase survival following induction of sepsis by injection of TNFα—a principal hyper-inflammatory cytokine produced in both LPS and CLP sepsis models. Notably, even when administered 2 hours after sepsis induction, TRPV4 channel antagonists provided significant protection against LPS-induced lethality, implying a longer-term protective effect on cytokine levels after the initial phase of the pathogenesis of sepsis. In support of this interpretation, we found that peritoneal leukocytes obtained from mice treated with LPS+GSK219 and incubated overnight *in vitro* displayed a reduction in TNFα and IL-6 production compared with those obtained from LPS-treated mice (Suppl. Fig. 3), despite only being exposed to the TRPV4 antagonist for a very short period of time *in vivo*.

The increased levels of inflammatory cytokines in sepsis impact vascular endothelial cells and contribute to their dysfunction. Cytokines released during endotoxemia have been shown to induce the expression of adhesion molecules, such as E-selectin and ICAM-1^44^. An increase in plasma adhesion molecules can be directly related to activation and/or dysfunction of the endothelium. Endothelial activation promotes rolling and firm adherence of neutrophils and platelets, and ultimately results in their accumulation in organs. This accumulation eliminates infectious agents and is thus partly beneficial, but it may also exacerbate tissue damage through the production of inflammatory mediators. Moreover, there is a close relationship between plasma levels of adhesion molecules and the consequences of sepsis. In human sepsis, higher plasma levels of E-selectin and/or ICAM-1 correlate with greater numbers of organs damaged, severity of sepsis, and mortality^45^,^46^. In animals, genetic or pharmacological blockade of ICAM-1 and PECAM-1 protects against endotoxin shock^47^,^48^, and a decrease in ICAM-1 and E-selectin expression correlates with decreased neutrophil infiltration in several organs (lung, liver, kidney) and a decrease in tissue damage^49^. High levels of PAI-1 and thrombomodulin are indicators of coagulation activation and a procoagulant endothelium in sepsis^50^,^51^. This procoagulant state contributes to multiple organ dysfunction syndrome in sepsis since it favors fibrin deposition in the microvasculature and contributes to hypoxygenation of tissues. In support of this, PAI-1 levels in septic patients have consistently been found to correlate with worsened outcome and the severity of multiple organ dysfunction syndrome^50^. We found that LPS-induced sepsis caused an increase in proMMP-9 and P-selectin, regardless of TRPV4 antagonist treatment. In contrast, LPS-induced increases in the adhesion molecules, ICAM-1 and PECAM-1, and the anticoagulant, thrombomodulin, were reduced by TRPV4 antagonist treatment. Furthermore, LPS-induced sepsis reduced the level and frequency of Ca^2+^ events (TRPV4 sparklets and IP3-mediated Ca^2+^ pulsars) in the endothelium, as well as the vasodilator response to the muscarinic receptor agonist carbachol – both of which are indicators of endothelial dysfunction and dysregulation of blood flow^22^,^29^,^31^. Finally, in LPS-induced sepsis, we observed an increase in hydraulic conductivity across the endothelium in mesenteric arteries, an indication of increased permeability that could potentially lead to edema formation. Importantly, TRPV4 channel antagonism reduced or abolished all of these deleterious vascular effects of LPS-induced sepsis. These results suggest that the reduction in hyper-inflammatory cytokines by TRPV4 blockade attenuates endothelial dysfunction, thereby protecting against sepsis-associated pathophysiological processes that lead to a proinflammatory and procoagulant state of the endothelium, impaired regulation of vascular tone and increased permeability, leading to hypotension, hypoperfusion and edema formation, which ultimately cause organ dysfunction and death.

Macrophages are the main contributors to cytokine production in sepsis^52^. Because the broad range of cytokines suppressed by TRPV4 channel blockade resembles the repertoire of cytokines produced by macrophages, we speculate that TRPV4 channel antagonists decreased hyper-inflammatory cytokine levels during sepsis development by blocking macrophage TRPV4 channels. In support of this possibility, TRPV4 channels are expressed in macrophages, regulate Ca^2+^ influx^36^,^37^,^38^, and have been implicated in the production of proinflammatory cytokines^32^. Moreover, blocking TRPV4 channels protects against the development of acute lung injury and heart failure by reducing pulmonary edema and inflammation^32^,^33^,^36^.

Sepsis-induced death is associated with organ dysfunction, such as acute lung injury, acute respiratory distress syndrome, liver failure, and acute kidney injury^53^. Surprisingly, we found no evidence of lung injury after LPS injection, as evidenced by the absence of significant changes in bronchoalveolar lavage fluid protein or leukocytes, or the presence of DIC in lungs (Suppl. Fig. 4). However, LPS injection increased AST, ALT, creatinine and BUN levels, indicating liver and kidney injury (Suppl. Fig. 1). TRPV4 channel blockade had no effect on the levels of creatinine or markers of liver injury after LPS injection and only moderately reduced BUN levels (Suppl. Fig. 1). Given the minor effect of TRPV4 antagonists on BUN and the absence of lung injury in this model, these results collectively suggest that the protective effects of TRPV4 blockade are not caused by a reduction in organ damage to lung, liver, or kidney. Whether TRPV4 blockade protects against cardiac failure—a common cause of death in sepsis^54^—remains to be investigated.

A recent study using cell therapy to target a broader range of sepsis mechanisms found that administration of fibroblastic reticular cells as much as 16 hours after LPS- or CLP-induced sepsis in mice decreased hyper-inflammatory cytokine levels and increased survival^55^, suggesting that targeting the hyper-inflammatory response as a whole may show more promising results than activated protein C and anti-TNFα antibody therapy in the treatment of sepsis^13^,^14^,^15^,^16^. Our demonstration that TRPV4 channel antagonism increases survival in model animals by reducing the production of inflammatory cytokines during the hyper-inflammatory phase of sepsis, thereby reducing the pro-inflammatory and pro-coagulant state of the endothelium and preserving endothelial-dependent dilation and integrity, reinforces this concept. Although these findings point to a crucial role for TRPV4 channel activity in the development of sepsis, the specific site at which TRPV4 antagonists act to suppress cytokine release *in vivo* remains unidentified. However, our results suggests that TRPV4 channels of interest reside upstream of the surge in cytokines, and likely involve the engagement of macrophage TRPV4 channels. Supporting this, a recent study suggests that macrophages TRVPV4 channels regulate the LPS-stimulated macrophage phagocytosis^38^. Our results argue against a central role of TRPV4 channels in cell types (e.g. endothelial) downstream of the elevation in TNFα and other cytokines. In support of this, a recent study found no protective effect of TRPV4 channel antagonist HC067 on hemodynamic parameters over a 24-hour period in a mouse LPS (12.5 mg/kg, i.v.) endotoxemia model^56^. Furthermore, knockout of the TRPC6 channel was recently reported to protect against LPS-induced lung permeability, inflammation and injury, suggesting a more general role for members of the TRP family in inflammatory responses. Unlike the case for TRPV4 antagonist-mediated protection against sepsis, this effect was attributed to disabling of TRPC6 in the vascular endothelium^57^. The TRPV4 channel antagonists used in our study have minimal adverse effects *in vivo* in animal models^33^,^39^, supporting their potential as agents to treat sepsis. From a clinical perspective, it should be carefully considered when to use future TRPV4 antagonists as a treatment option. Based on the concepts from Oberholzer *et al*.^58^, or Osuchowski *et al*.^59^, the evolution of the systemic immunoinflammatory response in sepsis may move from a hyper-inflammatory to a compensatory anti-inflammatory response or be a constant mixed anti-inflammatory response syndrome. Our data suggest that treatment with TRPV4 antagonists would potentially be more beneficial in a hyper-inflammatory phase of sepsis compared to a phase of a compensatory anti-inflammatory response or a mixed anti-inflammatory response. Collectively, our results suggest that TRPV4 channel antagonists may be useful not only in the treatment of sepsis, but also in pathological settings that involve a strong inflammatory response, such as organ transplantation, autoimmune disease, and allergy.

## Methods

### Animal procedures

Animal procedures and methods used in this study were approved by the University of Vermont Institutional Animal Care and Use Committee and performed in accordance with the National Institutes of Health policy on the care and use of laboratory animals. Wild-type C57BL6/J and TRPV4−/−21 mice were used in this study.

### LPS-, TNFα-, and CLP-induced sepsis

The LPS sepsis model was created by administering a single intraperitoneal (i.p.) dose of LPS (50 mg/kg, O55:B5; Sigma-Aldrich, St Louis, MO, USA), and the TNFα sepsis model was created by intravenous (i.v.) injection of TNFα (1 mg/kg) in the tail vein. The CLP procedure was done as previously described^40^. Briefly, Male C57BL/6 mice 10–16 weeks old were anesthetized with isoflurane, and the anterior abdominal wall was shaved. After a midline laparotomy, the cecum was exposed, ligated below the ileocecal valve without causing intestinal obstruction, and then punctured twice with a 21-guage needle. A small quantity of intestinal content was extruded to ensure hole patency. The cecum was then placed back in the peritoneal cavity, and the abdominal wall was closed with sutures and metal clips. Following the CLP procedure, mice were administered buprenorphine (0.03 mg/kg, i.p.) every 6 hours. Mice were divided into different groups, and the respective groups were treated with or without GSK219 (1 mg/kg, i.p.) or HC067 (10 mg/kg, i.p.) as indicated in the text. All mice subjected to LPS, TNFα, or CLP treatment were included in this study. Vehicle for LPS and TNFα was normal saline solution (NaCl 0.9%) and vehicle for GSK219 and HC067 was DMSO.

### Experimental design

For *ex vivo* investigations of the mechanisms involved in the protection against sepsis by TRPV4 channel blockade, mice were divided into the following groups based on the results of survival studies with LPS: control, 50 mg/kg (i.p.) LPS only, and 50 mg/kg (i.p.) LPS + 1 mg/kg (i.p.) GSK219 (administered 1 hour prior to LPS injection). Three and 18 hours after LPS injection, the following analyses were performed: (1) Blood was analyzed for cytokine levels, markers of activated endothelium, liver and kidney injury markers, and protein levels. (2) Bronchoalveolar lavage fluid was analyzed for leukocytes and protein, and lungs were isolated and fixed for determination of DIC. (3) Peritoneal lavage fluid was analyzed for cytokine levels. (4) Basal vascular endothelial Ca^2+^ events; TRPV4-mediated- and IP_3_R-mediated Ca^2+^ events, were imaged in isolated mesenteric arteries, and endothelium-dependent vasodilation and permeability were determined.

### Blood collection and assessment

Blood (150–200 μl) from anesthetized mice was collected into a heparinized needle and syringe by cardiac puncture and analyzed for blood cell composition (erythrocytes, platelets, and leukocytes) using an Advia 120 Hematology System (Bayer HealthCare, Leverkusen, Germany). Additional blood was then collected in the absence of anticoagulant and transferred to serum separator tubes (BD Biosciences), after which serum was collected and frozen. Thawed serum was diluted 1:4 in assay buffer, and cytokines were measured using a magnetic Luminex-based 23-plex mouse cytokine assay (Bio-Plex; Bio-Rad, Hercules, CA, USA), a 7-plex mouse cardiovascular disease panel, and metabolic panel (Milliplex, Millipore, Billerica, MA, USA), according to manufacturers’ instructions. Data were acquired using the Bio-Rad Bio-Plex suspension array system and Bio-Plex Manager 6.0 software. The fluorescence intensity of the background was subtracted from the values for each sample, standard, or control for each specific bead. Standard curves were generated from 4-fold serial dilutions of standards provided in the Bio-Plex or Milliplex kits and analyzed using a 5-place logistic regression of standards within 70–130% of the expected values. Upper levels of quantitation and lower levels of quantitation were calculated using Bio-Plex Manager software.

### BAL collection and processing

Lungs were lavaged with 1 mL Dulbecco’s phosphate-buffered saline (DPBS; Sigma-Aldrich, St. Louis, MO, USA), and the resulting BAL fluid was centrifuged and collected into separate tubes. Total cells in the pellet were resuspended in PBS and enumerated by counting with an Advia 120 Hematology System (Bayer HealthCare). Differential analyses were performed by hematoxylin and eosin (H&E) staining of cytospin preparations containing approximately 200 cells per slide. Protein in BAL fluid, an assessment of lung damage, was measured using the Bradford assay (Bio-Rad).

### Peritoneal lavage and assessment

The peritoneal cavity of naïve or LPS-exposed mice was lavaged using 5 ml of DPBS containing 3% fetal bovine serum (FBS, Cell Generation, Ft. Collins, CO, USA). TNFα and IL-6 in peritoneal lavage fluid was analyzed from undiluted samples by ELISA (BD Biosciences, San Diego, CA, USA).

### Pressurized arteries

Mice were euthanized by i.p. injection of sodium pentobarbital (150 mg/kg) followed by a thoracotomy. Third-order branches of mesenteric arteries (~100 μm internal diameter at 80 mm Hg) were isolated into HEPES-physiological saline solution (HEPES-PSS) (10 mM HEPES, 134 mM NaCl, 6 mM KCl, 1 mM MgCl_2_, 2 mM CaCl_2_, 7 mM glucose, pH 7.4) and used intact for diameter and permeability studies or cut longitudinally and pinned to a Sylgard block with the endothelium facing up (*en face* preparation). Diameter measurements of pressurized arteries were performed as described previously^28^,^60^. Briefly, mesenteric arteries were dissected free of surrounding tissue and mounted on similar-sized glass pipettes in an arteriograph chamber (Living Systems Instrumentation, St. Albans, VT, USA). The arteries were then pressurized to 80 mm Hg for at least 45 minutes in 36 °C physiological saline solution (PSS) (119 mM NaCl, 4.7 mM KCl, 1.2 mM KH_2_PO_4_, 1.2 mM MgCl_2_, 2 mM CaCl_2_, 7 mM glucose, 24 mM NaHCO_3_, pH 7.4) constantly equilibrated with bioair of the following composition: 20% O_2_ and 5% CO_2_ in N_2_ as the balancing gas. Internal diameter was continuously monitored with a CCD camera and edge-detection software (IonOptix, Milton, MA, USA). All compounds were added to the perfusate (PSS), which was continuously recirculated through the arteriograph chamber. Arteries were treated with Ca^2+^-free PSS (119 mM NaCl, 4.7 mM KCl, 1.2 mM KH_2_PO_4_, 2 mM CaCl_2_, 7 mM glucose, 24 mM NaHCO_3_, 5 mM EGTA, pH 7.4) at the conclusion of each experiment to obtain maximal dilation. Myogenic tone was calculated as [(Diameter_Ca_^2+^_−free_ – Diameter_basal_)/Diameter_Ca_^2+^_−free_] X 100, where Diameter_basal_ is the basal diameter of the artery pressurized at 80 mm Hg. Dilation was expressed as [(Diameter_dilated_ – Diameter_basal_)/(Diameter_Ca_^2+^_−free_ – Diameter_basal_)] X 100, where Diameter_dilated_ is the diameter following addition of CCh.

### Determination of mesenteric artery permeability

Permeability measurements were performed as described previously^61^. Briefly, mesenteric arteries were isolated as described above with the proximal end mounted on a similar-sized glass pipette in the arteriograph chamber and the distal end tied off with a nylon suture. The arteries were then pressurized to 80 mm Hg for at least 45 minutes in 36 °C PSS constantly equilibrated with biological atmosphere mixed gas (5% CO_2_, 20% O_2_, balance N_2_). Intravascular pressure was maintained and measured using a servo controller and a peristaltic pump (Living Systems Instrumentation). Permeability of isolated mesenteric arteries was determined by monitoring the drop in intravascular pressure over time—a reflection of the flux of intracellular fluid across the vessel wall—for 40 minutes following disconnection of the pressure-servo controller and peristaltic pump from the transducer. The drop in intravascular pressure was recorded continuously during the experiment with continuous monitoring of internal diameter using a CCD camera and edge-detection software (IonOptix, Milton, MA, USA).

The hydraulic conductivity, Lp, which reflects the specific permeability of the vessel to water, is determined by *Eq. 1*,
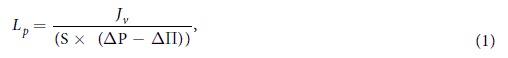
where S is the surface area of the vessel; *J*v is the trans-arterial volume flux;^62^,^63^ ΔP, the trans-arterial hydrostatic pressure difference, corresponds to the intravascular pressure used in these experiments; and ΔΠ is the trans-arterial osmotic pressure. Since the intraluminal fluid was identical to the fluid surrounding the cannulated vessel, ΔΠ was assumed to be 0. Therefore, in these experiments, equation for *L*p reduces to
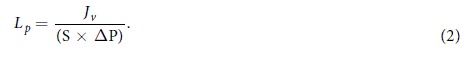


*J*v/*S* was calculated by *Eq. 3*,
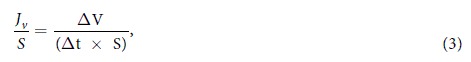
where ΔV represents the volume flux, and Δt is the time interval.

The surface area of the vessel *S* was calculated as
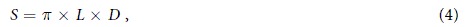
where L is the vessel length, D is the luminal diameter (*D*), and the vessels are assumed to be open-ended right cylinders.

Determination of *J*v/*S* and *L*p required conversion of the decrease in intravascular pressure per minute (mm Hg/min) to actual volume flux across the vessel wall (μm^3^). This was accomplished by creating a conversion graph of pressure versus volume. Because of the low volume of fluid flux at these low pressures, the volume of fluid for different decreases in pressure was determined.

### EC Ca^2+^ imaging

Ca^2+^ signals in endothelial cells were imaged with a Revolution Andor confocal system (Andor Technology, Belfast, UK) comprised of an upright Nikon microscope with a 60x, water-dipping objective (NA 1.0) and an electron-multiplying CCD camera, as we have described previously^28^,^31^,^60^. Briefly, images were acquired with Andor Revolution TL acquisition software (Andor Technology) at 30 frames/s. Bound Ca^2+^ was detected by exciting at 488 nm with a solid-state laser and collecting emitted fluorescence using a 527.5–49 nm band-pass filter. Experiments were performed at 36 °C. Endothelial cells in an *en face* preparation were loaded with Fluo-4-AM (10 μM) by incubating for 45 minutes at 30 °C in the presence of pluronic acid (2.5 mg/mL) before imaging and the effects of LPS and LPS+GSK219 on TRPV4-mediated- (sparklets)^28^ and IP_3_R-mediated (Ca^2+^ pulsars)^31^ Ca^2+^ events, were investigated.

### TRPV4 sparklets

TRPV4 sparklets was analyzed using custom-designed software written by Dr. A. D. Bonev^28^,^29^,^31^,^60^. Fractional fluorescence (F/F_0_) at a site was obtained by dividing the fluorescence of a region of interest (ROI) defined by a 1.7 μm^2^ (5 × 5 pixels) box positioned at a point corresponding to peak Ca^2+^ amplitude in the collected image by the average fluorescence of 10 images without activity from the same ROI. Area under the curve (AUC) for each opening was calculated using trapezoidal numerical integration ([F − F_0_]/F_0_ over time). Activity at a TRPV4 sparklet events site was calculated as the integrated AUC of all openings at that site within a 2-minute recording duration. The activity integrals from all sites in a field were added to obtain total activity for that field.

Cyclopiazonic acid (CPA, 30 μM, 15–20 minutes), which blocks the endoplasmic reticulum Ca^2+^-ATPase (SERCA), was included to eliminate Ca^2+^ pulsars, when measuring TRPV4 sparklets. Furthermore, TRPV4 sparklets were measured in the presence of 3 nM GSK1016790A, a TRPV4 channel agonist, to ensure a substantial baseline channel activity as previously reported^28^,^29^.

### Ca^2+^ pulsars

Local, stationary Ca^2+^ pulsars^31^, were measured offline by detecting an increase in the fractional fluorescence (F/F_0_) that was significantly above background (F/F_0_ > 1.2). F/F_0_ was evaluated in 9 × 9-pixel regions of interest in the collected image positioned at points corresponding to peak pulsar amplitude; F_0_ was obtained from the same region of interest in 10 images without activity. The kinetic properties of the Ca^2+^ pulsars were analyzed using our custom software^28^,^29^,^31^,^60^, and Ca^2+^ pulsar frequency was determined as number of events over time per field of view.

### Statistical analysis

Statistical calculations were performed using GraphPad Prism software (GraphPad Software, La Jolla, CA). Survival data were compared using the Mantel-Cox test. All other data were compared using the Kruskal-Wallis test. Statistically significant (p < 0.05) results by Kruskal-Wallis were further analyzed by Dunn’s multiple comparisons test. Randomization of animals to experimental groups and downstream assays or blinding of the investigators was not possible due to the visual difference of the treated animal groups and the technical layout of the multiple assays used in this study. Sample size was determined based on power analyses, in which the number of animals required to detect a 5% difference in mortality in the sepsis models would be revealed with 80% confidence.

## Additional Information

**How to cite this article**: Dalsgaard, T. *et al*. Pharmacological inhibitors of TRPV4 channels reduce cytokine production, restore endothelial function and increase survival in septic mice. *Sci. Rep.* **6**, 33841; doi: 10.1038/srep33841 (2016).

## Supplementary Material

Supplementary Information
