# Supplementary material for: Pharmacological inhibitors of TRPV4 channels reduce cytokine production, restore endothelial function and increase survival in septic mice
Source: Sci Rep. 2016 Sep 22;6:33841. doi: 10.1038/srep33841 (PMC5031985; doi:10.1038/srep33841)
Supplement: Supplementary Information [file srep33841-s1.pdf]

Title:

Pharmacological inhibitors of TRPV4 channels reduce cytokine production, restore endothelial function and increase survival in septic mice

Authors: Thomas Dalsgaard[1], Swapnil K. Sonkusare[1], Cory Teuscher[2], Matthew E. Poynter[3], Mark T. Nelson[1,4]*

Author affiliation: [1]Department of Pharmacology, College of Medicine, University of Vermont, Burlington, VT 05403, USA. [2]Department of Medicine, Immunobiology Program, University of Vermont, Burlington, VT 05403, USA. [3]Department of Medicine, Division of Pulmonary Disease and Critical Care, University of Vermont, Burlington, VT 05403, USA. [4]Institute of Cardiovascular Sciences, University of Manchester, Manchester M13 9NT, UK.

*Corresponding author: Mark T. Nelson, Department of Pharmacology, University of Vermont, Given B-333A, 89 Beaumont Avenue, Burlington VT 05405-0068, telephone: (802) 656-2500, email: mark.nelson@uvm.edu

Supplementary figures

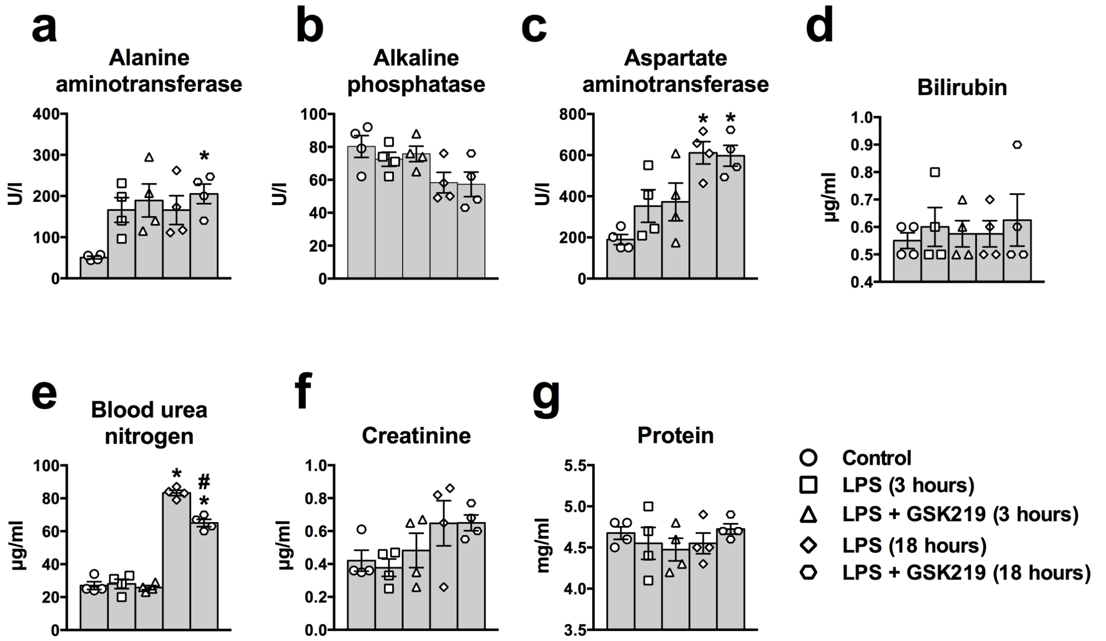

Supplementary figure 1. TRPV4 channel blockade moderately decreases BUN in LPS-induced sepsis in vivo. Serum markers of organ damage in mice treated with LPS only or LPS + GSK219 were measured 3 and 18 hours after LPS. In all cases, LPS and GSK219 were injected (i.p.) at doses of 50 mg/kg and 1 mg/kg, respectively; where used, GSK219 was administered 1 hour prior to LPS injection. (**a**) Alanine aminotransferase (ALT), (**b**) alkaline phosphatase, (**c**) Aspartate aminotransferase (AST), (**d**) bilirubin, (**e**) Blood urea nitrogen (BUN), (**f**) creatinine, and (**g**) Total blood protein. Data are expressed as means ± SEM (n = 4 mice/group; *p < 0.05 vs. control, #p < 0.05 vs. LPS treatment at the same time point, Kruskal-Wallis test with Dunn's multiple comparisons test).

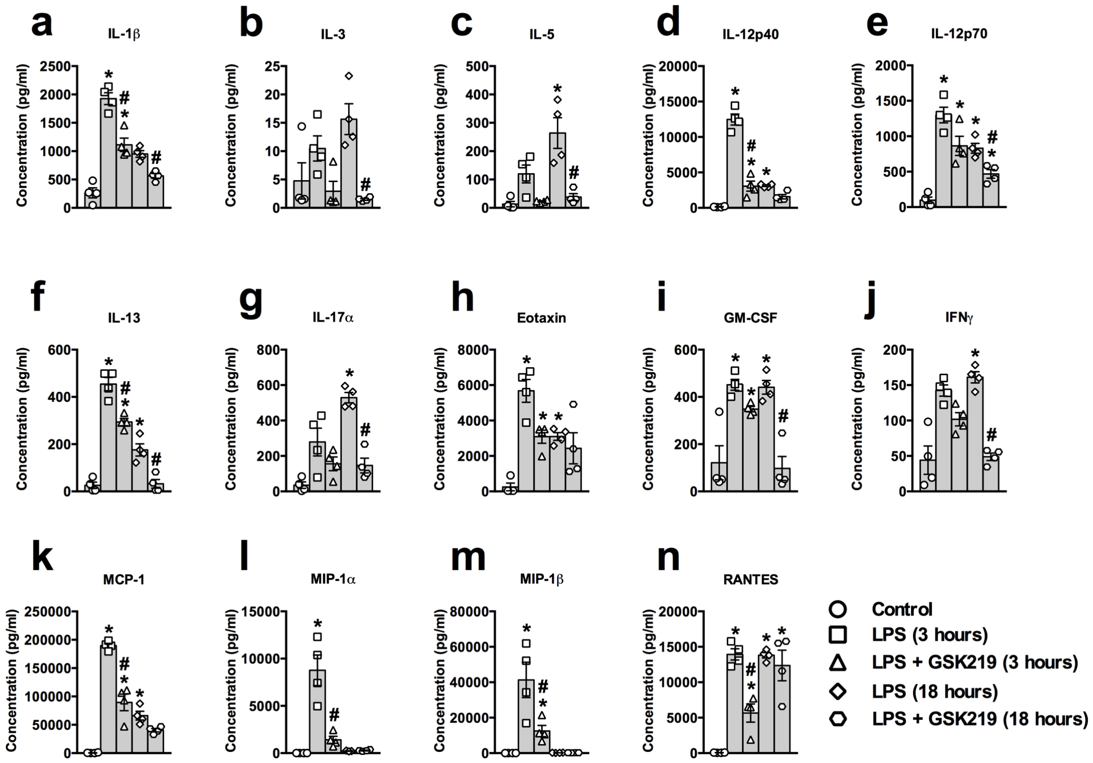

Supplementary figure 2. TRPV4 channel blockade reduces the concentration of blood cytokines in LPS-induced sepsis in vivo. Blood concentrations of (**a**) IL-1β, (**b**) IL-3, (**c**) IL-5, (**d**) IL-12p40, (**e**) IL-12p70, (**f**) IL-13, (**g**) IL-17α, (**h**) eotaxin, (**i**) GM-CSF, (**j**) IFNγ, (**k**) MCP-1, (**l**) MIP-1α, (**m**) MIP-1β, and (**n**) RANTES, 3 and 18 hours after injection of LPS only (50 mg/kg, i.p.) or LPS + GSK219 (1 mg/kg, i.p., injected 1 hour prior to LPS). Data are expressed as means ± SEM (n = 4 mice/group; *$p < 0.05$ vs. control, #$p < 0.05$ vs. LPS treatment at the same time point, Kruskal-Wallis test with Dunn's multiple comparisons test). GM-CSF, granulocyte macrophage colony-stimulating factor; IFNγ, interferon gamma; IL, interleukin; MCP-1, monocyte chemoattractant protein-1; MIP, macrophage inflammatory protein; RANTES, regulated on activation normal T cell expressed and secreted.

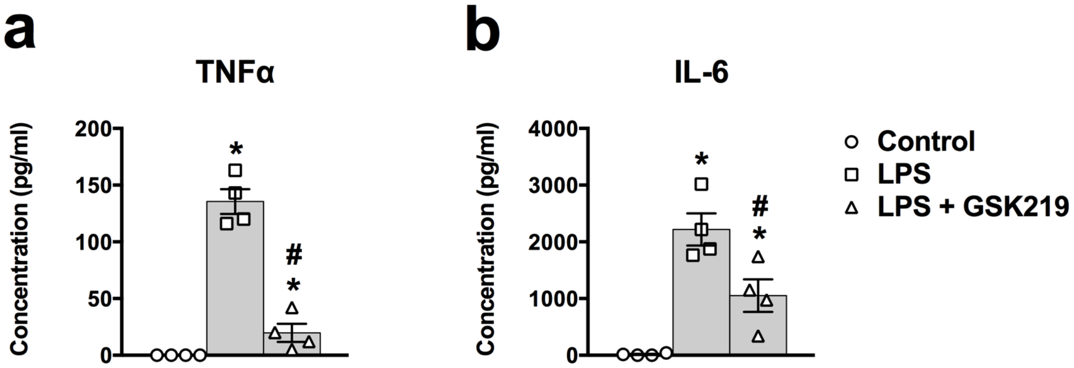

Supplementary figure 3. TRPV4 channel blockade reduces TNFα and IL-6 production from peritoneal leukocytes cultured in vitro following LPS-induced sepsis in vivo. Levels of (**a**) TNFα and (**b**) IL-6 in culture supernatants from peritoneal cells from mice treated with LPS only or LPS + GSK219. Peritoneal lavage was conducted 1 hour after injection of LPS or LPS + GSK219, and peritoneal cells were cultured for 16 hours in vitro to allow for secretion of TNFα and IL-6. LPS and GSK219 were injected (i.p.) at doses of 50 mg/kg and 1 mg/kg, respectively. Data are expressed as means ± SEM (n = 4 mice/group; *p < 0.05 vs. control, #p < 0.05 vs. LPS treatment at the same time point, Kruskal-Wallis test with Dunn’s multiple comparisons test).

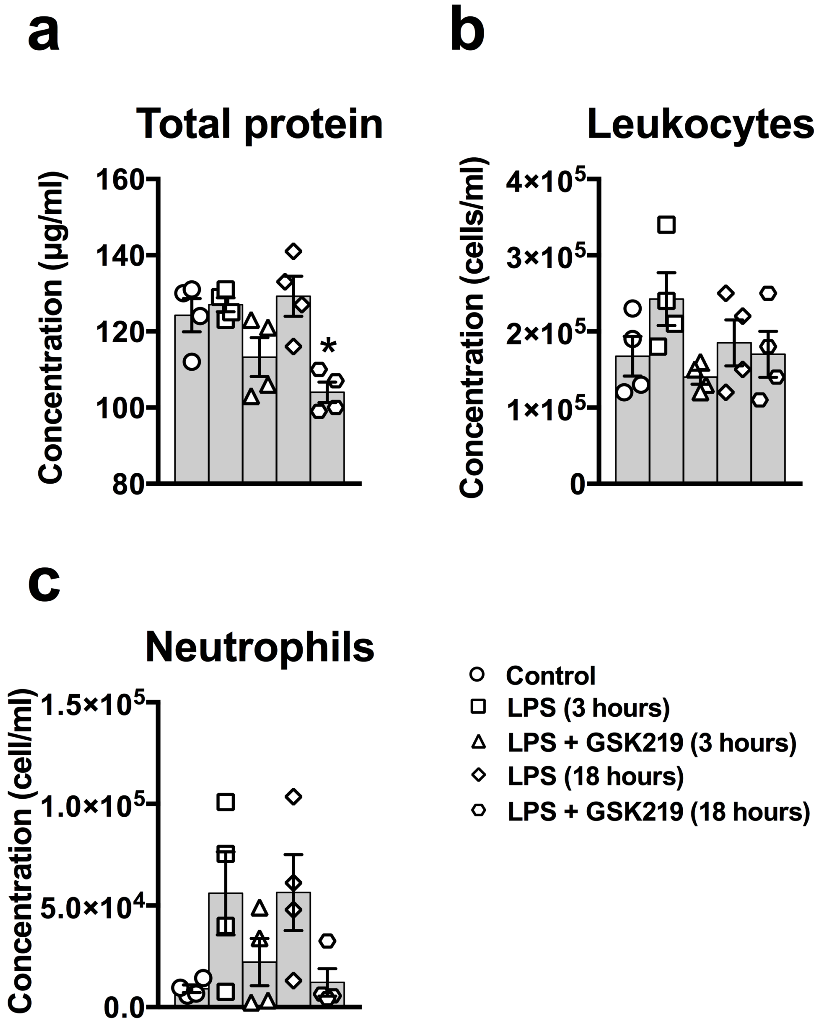

Supplementary figure 4. LPS-induced sepsis has no effect on protein, leukocyte, or neutrophil concentration in bronchoalveolar lavage fluid. Concentrations of (**a**) protein, (**b**) leukocytes and (**c**) neutrophils in bronchoalveolar lavage fluid collected 3 and 18 hours after injection of LPS only or LPS + GSK219. In all cases, LPS and GSK219 were injected (i.p.) at doses of 50 mg/kg and 1 mg/kg, respectively; where used, GSK219 was administered 1 hour prior to LPS injection. Data are expressed as means ± SEM (n = 4 mice/group; *p < 0.05 vs. control, #p < 0.05 vs. LPS treatment at the same time point, Kruskal-Wallis test with Dunn's multiple comparisons test).
